# Cysteinolic Acid Is a Widely Distributed Compatible Solute of Marine Microalgae

**DOI:** 10.3390/md19120683

**Published:** 2021-11-30

**Authors:** Simona Fenizia, Jerrit Weissflog, Georg Pohnert

**Affiliations:** 1 Bioorganic Analytics, Institute for Inorganic and Analytical Chemistry, Friedrich Schiller University Jena, Lessingstrasse 8, D-07743 Jena, Germany; simona.fenizia@uni-jena.de; 2 MPG Fellow Group, Max Planck Institute for Chemical Ecology, Hans-Knöll-Straße 8, D-07745 Jena, Germany; jweissflog@ice.mpg.de

**Keywords:** cysteinolic acid, osmoadaptation, osmoregulation, ectoine, DMSP, diatoms, phytoplankton, salinity, LC/MS analysis

## Abstract

Phytoplankton rely on bioactive zwitterionic and highly polar small metabolites with osmoregulatory properties to compensate changes in the salinity of the surrounding seawater. Dimethylsulfoniopropionate (DMSP) is a main representative of this class of metabolites. Salinity-dependent DMSP biosynthesis and turnover contribute significantly to the global sulfur cycle. Using advanced chromatographic and mass spectrometric techniques that enable the detection of highly polar metabolites, we identified cysteinolic acid as an additional widely distributed polar metabolite in phytoplankton. Cysteinolic acid belongs to the class of marine sulfonates, metabolites that are commonly produced by algae and consumed by bacteria. It was detected in all dinoflagellates, haptophytes, diatoms and prymnesiophytes that were surveyed. We quantified the metabolite in different phytoplankton taxa and revealed that the cellular content can reach even higher concentrations than the ubiquitous DMSP. The cysteinolic acid concentration in the cells of the diatom *Thalassiosira weissflogii* increases significantly when grown in a medium with elevated salinity. In contrast to the compatible solute ectoine, cysteinolic acid is also found in high concentrations in axenic algae, indicating biosynthesis by the algae and not the associated bacteria. Therefore, we add this metabolite to the family of highly polar metabolites with osmoregulatory characteristics produced by phytoplankton.

## 1. Introduction

Free-floating phytoplankton in the open ocean contribute substantially to primary production. Phytoplankton can be exposed to changing salinity levels due to vertical migration or transport within ocean currents [1]. Adaptation to the changing environment is thus a pre-requisite for the survival of the cells [1,2]. Changes in salinity that occur, for example, in coastal waters or sea ice represent an environmental stress factor that must be compensated by the unicellular algae of the phytoplankton [3]. Cells respond by production/uptake or degradation/exudation of compatible solutes. These polar organic compounds adjust the osmolality of the cells to that of the environment [4,5,6,7]. Dimethylsulfoniopropionate (DMSP) is a major compatible solute in many phytoplankton species. The amount of this sulfur-containing zwitterionic metabolite produced globally by marine organisms is estimated to be around two petagrams per year [8,9]. The enzymatic cleavage of this osmolyte results in the release of the volatile dimethylsulfide (DMS). This metabolite contributes significantly (30 teragram per year) to the global sulfur flux from the hydrosphere to the atmosphere [10,11,12,13]. Besides DMSP, many other highly polar metabolites that can serve as compatible solutes have been discovered in phytoplankton. Analysis of these metabolites has proven problematic because it is difficult to extract and enrich the small, polar metabolites. The introduction of a ZIC-HILIC separation protocol combined with highly sensitive MS analysis has enabled the systematic investigation of zwitterionic metabolites and other polar metabolites in marine microalgae and seawater [14,15,16,17]. The identification of dimethylsulfoxoniumpropionate (DMSOP) produced and released by phytoplankton is a remarkable demonstration of the validity of this analytical approach. DMSOP is produced by phytoplankton and is readily taken up by marine bacteria, confirming that this class of molecules supports bacterial heterotrophy in the ocean [11,18,19].

Besides sulfur-containing zwitterionic metabolites, marine organisms also produce nitrogen-containing compounds, such as glycine betaine (GBT) and homarine, with osmoregulatory and osmoadaptive properties [4]. The bacterial zwitterionic metabolite ectoine, for example, is also a common metabolite produced by marine phytoplankton and bacteria [7]. It can be taken up from bacteria by diatoms to fulfill physiological requirements and to compensate for osmotic stress [7].

Despite the considerable advances in the characterization of the phytoplankton metabolome, organic polar compounds are still not comprehensively analyzed [4,7,11]. A detailed analysis of sulfonates from cultured eukaryotic phytoplankton showed up to millimolar cellular concentrations. The analysis of the production and catabolism of this compound class revealed a tightly coupled microbial network based on sulfonates. Sources and sinks were deduced from genomic data, expression analysis and mass spectrometric evidence [20].

Our study aims to expand our knowledge of the metabolome of marine algae, focusing on the production and regulation of polar compounds produced under osmotic stress conditions. For this purpose, we selected the diatom *Thalassiosira weissflogii* as a study organism. It is a centric marine diatom, ubiquitously distributed in the oceans [21]. *T. weissflogii* has been utilized previously for several studies on the osmoadaptation of marine phytoplankton [22,23]. It was also used as a model organism for the investigation of diatom responses to different types of environmental stress, such as seawater acidification [24], temperature and light changes [25] and anoxia [21]. Interestingly, *T. weissflogii* does not produce the ubiquitous osmolyte DMSP but compensates this lack by uptake of the metabolite from the surrounding water [23]. We detected and quantified cysteinolic acid under osmotic stress conditions in this alga and undertook a survey of its prevalence in other members of the phytoplankton. We observed a significant contribution of this metabolite to the osmoadaptation of microalgae during short- and long-term environmental salinity changes. Therefore, cysteinolic acid can be included among the phytoplankton-derived “compatible solute”.

## 2. Results and Discussion

Xenic and axenic cultures of the diatom *Thalassiosira weissflogii* were grown under standard culture conditions of 35 Practical Salinity Units (PSU, g NaCl kg^−1^ seawater) and under increased salinity of 50 PSU to cover the effect of a substantial salinity increase according to previous studies [7,26,27]. Using ultra-high-pressure liquid chromatography and high-resolution mass spectrometry (UHPLC-MS) [4], we mined for uncharacterized highly polar metabolites potentially involved in osmoregulation. Up-regulation under increased salinity was selected as a criterion in a metabolic profiling approach. Among the up-regulated masses that could not be assigned to common and fully structurally proven metabolites, we detected [M + H]^+^ = 156 with a characteristic sulfur isotope signature. It was characterized as cysteinolic acid, a compound with osmoregulatory properties.

### 2.1. Identification of Cysteinolic Acid in the Diatom Thalassiosira weissflogii

**Figure 1:**
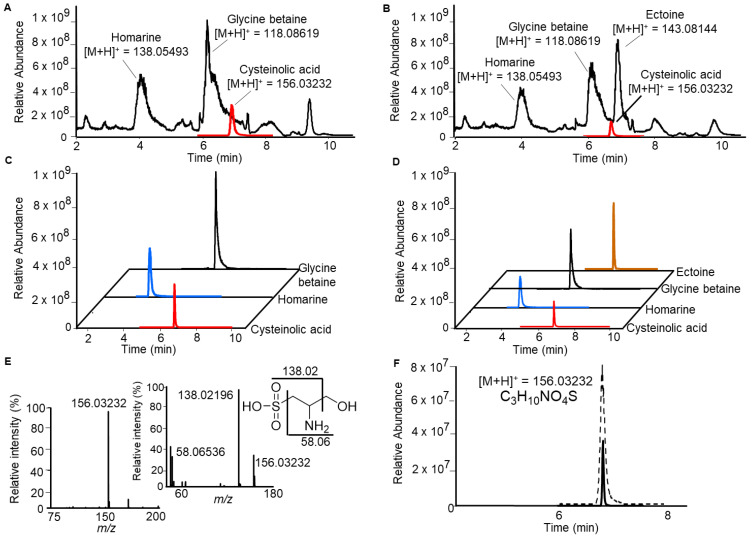
Chromatographic separation of highly polar and zwitterionic metabolites in the diatom *Thalassiosira weissflogii* CCMP1336 (axenic cultures, **A**), RCC76 (xenic cultures, **B**), using ultra-high-pressure liquid chromatography (UHPLC) with detection by electrospray ionization mass spectrometry. The total ion current is shown in black. The identity of the metabolites glycine betaine, homarine and ectoine was assigned according to previous studies. The ion trace of cysteinolic acid (solid red line) is shown at a fivefold magnification in (**A**) and at a tenfold magnification in (**B**). The extracted ion chromatograms for the selected zwitterions are shown in (**C**, axenic culture) and in (**D**, xenic culture). The MS and MS/MS spectra of cysteinolic acid are shown in (**E**), and fragmentation is indicated in the inserted structure. In (**F**), the solid black line is the UHPLC profile monitoring the ion trace of *m/z* = 156.03232 ± 0.0005% of the methanol extract of cultures of *T. weissflogii*; the dashed black line is the same extract treated with synthetic cysteinolic acid in roughly equal amounts to confirm structural identity by co-elution.

Figure 1 shows the UHPLC-HRMS chromatographic profile obtained from methanolic extracts of xenic and axenic cultures of *T. weissflogii* (the respective strains used were RCC76 and CCMP1336). This diatom does not produce quantifiable amounts of DMSP, but other major zwitterionic metabolites, such as homarine, glycine betaine and ectoine, are detectable [7,23]. The identities of these osmolytes were verified by co-injection either with commercially available standards (glycine betaine and ectoine) or with the corresponding standard synthesized in our laboratory (homarine) according to a published procedure [4].

Analysis of the chromatogram and the HRMS spectra in positive mode revealed a peak with *m/z* of the [M + H]^+^ = 156.03232, corresponding to the molecular formula C_3_H_10_NO_4_S (calculated: [M + H]^+^ = 156.03251). This initially caught our attention due to the isotope pattern with a M + 1.9958 signal, characteristic for sulfur-containing metabolites. The incorporation of ^13^C from ^13^CO_2_ confirmed its biosynthetic origin. The fragmentation pattern of this peak, analyzed by tandem mass spectrometry (MS/MS), showed a fragment ion *m/z* 138.02196, attributed to the loss of H_2_O, and a second one with *m/z* 56.04979 attributed to the loss of water and the sulfonic group (Figure 1). By comparison with the online database METLIN and using the tools Sirius v4.0.1, and CSI:FingerID [28,29], the signal was initially attributed to cysteinolic acid, whose identity was proven by co-injection of the algal extract with synthetic cysteinolic acid. Both exact mass and retention time matched the standard and co-injection confirmed the identity of the metabolite (Figure 1F).

**Figure 2:**
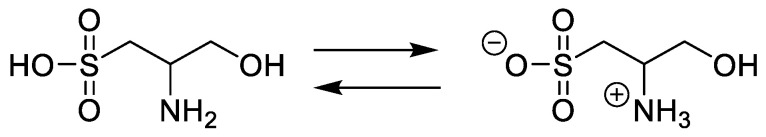
All neutral (**left**) and zwitterionic (**right**) forms of cysteinolic acid.

Cysteinolic acid (2-amino-3-hydroxy-1-propanesulfonic acid) belongs to the class of sulfonates common in aquatic systems. Unlike the zwitterions, such as homarine, DMSP, glycine betaine, DMSOP and gonyol, cysteinolic acid can be present in a neutral form as depicted in Figure 2.

In 1957, Wickberg et al. reported the isolation and identification of this C3-sulfonate in the red alga *Polysiphonia fastigiata* and thereby expanded the knowledge about sulfonates in algae that was limited to taurine and taurine derivates [30]. Other studies reported cysteinolic acid in brown algae [31] and in the freshwater diatom *Navicula pelliculosa* where it occurs together with the structurally related 2,3-dihydroxypropane-1-sulfonate (DHPS). In this alga, it is one of the major sulfonic acids and plays a role as sulfur reservoir during sulfur starvation [32,33]. Based on the incorporation of radioactive sulfate, it was concluded that cysteinolic acid accounts for ca. 11% of the total soluble ^35^S. Besides these early reports on freshwater diatoms, little is known about the distribution, abundance and role of cysteinolic acid in marine microalgae. Recent studies have focused on DHPS and have pointed out the presence of sulfonate transport and catabolism genes in terrestrial and marine bacteria and microalgae [20,34]. In an untargeted metabolomics approach, evidence for the production of cysteinolic acid in marine diatoms and a haptophyte was given based on MS/MS data similar to those reported above, but the final structural confirmation, as well as information about its concentration and physiological role, remained open [20]. It was speculated that such a highly polar metabolite could contribute to osmoadaptation and that this compound class could also be involved in the balance of redox reactions [20].

DHPS and other osmolytes, particularly DMSP, betaine and proline, increase with salinity in the diatom *T. pseudonana*. The high cellular concentration (mM) of sulfonates produced by marine phytoplankton supports the notion that this class of metabolites plays an important role in the environment and makes a substantial contribution to global sulfur and carbon cycles likely [20,34,35].

### 2.2. Salinity-Dependent Changes in Cysteinolic Acid Concentration in T. weissflogii

To verify if cysteinolic acid contributes to the intracellular osmotic balance in marine diatoms, *T. weissflogii* was grown at low (35 PSU) and high (50 PSU) salinity, according to protocols of previous studies [7,26]. For short-term salinity stress experiments, cells were grown at 35 PSU to the late exponential phase, transferred to 50 PSU, and analyzed 24 h after this transfer. Long-term salinity stress was achieved by growing cells at 50 PSU over two generations. Tests were performed on both axenic and xenic cultures. *T. weissflogii* cells were harvested by filtration on GF/C filters, which also removes most of the bacteria present in xenic cultures [7].

**Figure 3:**
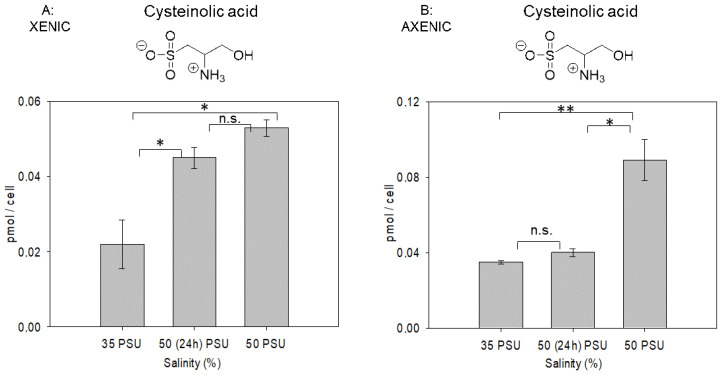
Intracellular amounts of cysteinolic acid in xenic (**A**) and axenic (**B**) cultures of *T. weissflogii* grown under different salinity regimes. The label 35 PSU indicates that cultures were maintained constantly at this salinity; 50 PSU (24 h) indicates that cultures grown at 35 PSU were transferred into a medium of 50 PSU and analyzed 24 h after the transfer; 50 PSU indicates cultures grown for two generations at this elevated salinity. Concentrations are normalized per cell; error bars represent standard deviation (biological replicates, *N* = 3). Statistical analysis is based on one-way ANOVA with a Tukey test for multiple comparison procedures. * *p* ≤ 0.05, ** *p* ≤ 0.01, n.s. not significan). Note the different scales of the *y*-axes.

Quantification of cysteinolic acid revealed that it is also a substantial osmolyte in this diatom with intracellular concentrations reaching the 100 fmol/cell level (Figure 3). This is lower compared to ectoine, homarine and glycine betaine that reach pmol/cell concentrations [7].

After short-term salinity stress in xenic cultures, the concentration of cysteinolic acid increased by ca. 2-fold compared to the concentration under 35 PSU conditions. A 2.4-fold increase was observed after long-term stress (Figure 3). In axenic cultures, compared to the xenic, the intracellular content of cysteinolic acid was 1.5-fold higher at 35 PSU, and it did not increased significantly during the short-term salinity stress, but increased by 2.6-fold during the long-term stress (Figure 3). In axenic cultures, the higher amount of cysteinolic acid might compensate for the lower amount of ectoine (observed in a previous study [7]). Ectoine is predominantly supplied by bacteria and taken up by the algae, and thus reaches lower concentrations in axenic cultures [7]. This is not the case for cysteinolic acid, which is higher in axenic studies. It is thus produced by the algae and might be consumed or converted by associated bacteria in xenic cultures. These regulations support a contribution of the microbial community to the physiology of an algal cell and the existence of a coupled microbial metabolic network between eukaryotic phytoplankton (sulfonate producers) and heterotrophic bacteria (sulfonate consumers, ectoine producers) [20,35,36,37].

### 2.3. Cysteinolic Acid in Other Microalgae

With the purpose of investigating the distribution of cysteinolic acid in phytoplankton, the metabolome of several species of microalgae was screened and the intracellular amounts of cysteinolic acid were compared to those of other zwitterionic osmolytes [7,11]. The amount of cysteinolic acid in all the investigated species was obtained by integration of the correspondent signal in each chromatogram and an external calibration curve prepared with three repeated measurements before each batch analysis. The concentration was then normalized to cell counts and cellular volume. The concentrations of cysteinolic acid can reach levels in the same range of those of DMSP, which is a dominant zwitterionic osmolyte in many species (Table 1) [11,38]. It is even the most abundant osmolyte quantified in this study in the dinoflagellate *Amphidinium carterae*. The intracellular concentrations of cysteinolic acid are in the same range as those of DHPS, a major cytosolic diatom component that supports bacterial growth [20]. 

The abundance and the regulation under different salinity regimes suggest a contribution of this zwitterionic metabolite to the osmoregulation of different plankton phyla. It adds to the family of zwitterionic metabolites as a major component in the algal cells that support osmoadaptation. The abundance of cysteinolic acid also supports the notion of sulfonates as a valuable target for tracking microbial interactions in the sea. As revealed by Durham et al. [20], sulfonate metabolism in the marine biosphere is widely distributed and highly complex. Our result adds a new algal metabolite to this family by providing full structural proof and confirms eukaryotic phytoplankton as major sulfonate producers [18]. We also show its contribution to salinity adaptation. Since cellular content will eventually also contribute to the dissolved organic carbon and dissolved organic sulfur in the ocean that can be used by other microorganisms of the plankton, the consequence of this additional metabolite on the microbiome functioning should be investigated. In agreement, genes connected to the uptake and metabolism of dissolved organic sulfur are also abundant.

## 3. Materials and Methods

The results in this work arise from an in-depth analysis of an experiment reported by Fenizia et al. [7]. There, the full experimental details are given. Here we only outline the methods for cultivation and extraction briefly. The full methods for structure elucidation and quantification are given here.

### 3.1. Cultivation of Microalgae

Xenic and axenic cultures of *Thalassiosira weissflogii* (RCC76, Roscoff Culture Collection, Roscoff, France; CCMP 1336; Provasoli-Guillard National Center for Marine Algae and Microbiota, East Boothbay, ME, USA) were cultivated as standing cultures in artificial seawater medium according to Maier and Calenberg [40] at 14 °C ± 2 °C. A 14:10 light–dark cycle with 40 μmol photons m^−2^s^−1^ between 400 and 700 nm light was used. Cultures were grown to the exponential phase; 2 mL was diluted 20-fold with fresh medium and cultivated again to the exponential phase before the start of the experiments. Axenicity was checked by microscopy and by plating aliquots of each culture on marine broth agar plates.

### 3.2. Salinity Treatment

For long-term salinity stress treatments, cultures of *T. weissflogii* were grown in artificial seawater with a normal salinity of 35 Practical Salinity Units (PSU), and in artificial sea water where the salinity was adjusted to 50 PSU [7]. For a short-term salinity stress test, cultures of the diatom were grown in 35 PSU artificial seawater to the exponential phase and, 24 h before the extraction, 5 mL of a sterile filtered 2.65 M solution of NaCl was added to 35 mL cultures in order to reach a final salinity of 50 PSU.

### 3.3. Cell Counting and Size Measurement

To determine cell densities, 50 μL of culture was analyzed using a BD Accuri^TM^ C6 flow cytometer (BD Biosciences, East Rutherford, NJ, USA) as described in [7]. Pictures for cell size measurements were taken with a Leica DFC280 microscope (Leica, Wetzlar, Germany) using a Nikon DS-U3 camera (Nicon, Tokyo, Japan). Pictures of 50 randomly selected cells for every salinity were evaluated. Calculations of the average cell volumes were based on a corresponding geometric shape, as reported by Hillebrand et al. [7,39].

### 3.4. Sample Preparation

Diatom cells were harvested in the late exponential growth phase by filtration of 30 mL culture under a reduced pressure of 500 mbar using GF/C-grade microfiber filters (Sigma-Aldrich, Deisenhofen, Germany), followed by washing through a vacuum filtration of 90 mL of artificial sea water [7]. Filters were immediately transferred into 4 mL screw cap glass vials containing 500 μL of methanol, while another portion of 500 μL of methanol was added directly on the filter. Samples were manually shaken three times and, after 30 min at room temperature, stored at −20 °C. For ultra-high-pressure liquid chromatography high-resolution mass spectrometry (UHPLC-HRMS) analysis, 50 μL of each extract was diluted with 100 μL of acetonitrile and water (9:1 *v/v*). After centrifugation (5 min, 4.500× *g*) the supernatant was submitted to UHPLC-HRMS analysis.

### 3.5. UHPLC/HRMS-Equipment and Settings

Analytical separation and quantification were performed on a Dionex^TM^ UltiMate^TM^ 3000 system (Thermo Fisher Scientific, Dreieich, Germany) equipped with a SeQuant ZIC-HILIC column (5 μm, 2.1 × 150 mm, SeQuant with guard column, Merck, Darmstadt, Germany). Mass spectra were recorded on a Q-Exactive^TM^ Plus Orbitrap mass spectrometer (Thermo Fisher Scientific, Dreieich, Germany). The electrospray ionization conditions are given in [7]. The column temperature was set to 25 °C. Mass measurements were performed in the HESI-positive mode, full scan mode from 75 to 200 *m/z*, at a resolution of 70,000. For qualitative MS/MS analysis, the collision energy was set to 35 V and data were collected in DIA (Data Independent Acquisition) mode.

### 3.6. Osmolyte Analysis

The eluent consisted of high-purity water with 2% acetonitrile and 0.1% formic acid (solvent A) and 90% acetonitrile with 10% water and 5 mmol L^−1^ ammonium acetate (solvent B) [15]. The flow rate was set to 0.6 mL min^−1,^ and a linear gradient was used for separation with 100% solvent B (2 min), 60% B (11 min), 20% B (11.8 min), 20% B (14.9 min), 100% B (15 min) and 100% B (18 min) at 25 °C. Identification by co-injection of cysteinolic acid was carried out after addition of synthetic cysteinolic acid to the sample. For quantification, a calibration curve of the synthetic standard was recorded in the seawater medium also used for algal culturing. The purity and concentration of the synthetic standard were verified by ^1^H-NMR. The calibration curve (*n* = 3) for the area of the molecular ion of cysteinolic acid was *y* = 1.27 × 10^7^ *x* with *r* = 0.9734, limit of detection (LOD) = 2.17 μM in the medium and limit of quantification (LOQ) = 7.15 μM in the medium. Related to ectoine, the calibration curve (*n* = 3) for the area of the molecular ion was *y* = 2.96 × 10^8^ *x* with *r* = 0.9970, limit of detection (LOD) = 0.33 μM and limit of quantification (LOQ) = 1.08 μM; for homarine, the calibration curve (*n* = 3) for the area of the molecular ion was y = 5.26 × 10^8^ *x* with *r* = 0.9931, limit of detection (LOD) = 0.04 μM, limit of quantification (LOQ) = 0.12 μM; for glycine betaine, *y* = 1.59 × 10^8^*x* with *r* = 0.9364, limit of detection (LOD) = 0.18 μM and limit of quantification (LOQ) = 0.58 μM. The calibration curve (*n* = 3) for the area of the molecular ion of DMSP was *y* = 8.28 × 10^7^ *x* with *r* = 0.9878, limit of detection (LOD) = 0.07 μM and limit of quantification (LOQ) = 0.23 μM. MS and MS/MS data are deposited in https://edmond.mpdl.mpg.de/imeji/collection/wP7a_7LNwS3DMf (accessed on 25 November 2021).

### 3.7. Synthesis of Cysteinolic Acid 

The synthesis was carried out using a slightly modified protocol of that of Xu et al. [41]. If not otherwise indicated, all solvents and chemicals were obtained from Sigma (Sigma, Deisenhofen, Germany) and used without further purification. In a 100 mL round-bottom flask, 200 mg (1.98 mmol) of methyl aziridine-2-carboxylate (TCI Co. Ltd., Tokyo, Japan) was dissolved in 20 mL of deionized water, and sodium borohydride (150 mg, 3.96 mmol) and lithium chloride (168 mg, 3.96 mmol) were added to the solution. After the mixture was stirred overnight at room temperature, sodium bisulfite (412 mg, 3.96 mmol) was added portion-wise under stirring. The resulting solution was stirred for another 24 h at room temperature. After this time, the complete reaction mixture was passed through a column of Amberlite IR-120 (H^+^ form) and then of Amberlyst A21 (free base form) using deionized water to rinse the columns. The pooled eluate fractions were concentrated under reduced pressure back to a volume of ~20 mL before being passed through a column of Dowex 1 × 8 (acetate form, prepared from chloride form). The eluate was evaporated to dryness under reduced pressure, and the residue was recrystallized from EtOH/H_2_O (3:1, *v*/*v*) three times to give (**1**) (80 mg, 0.51 mmol, 26%) as a white solid, which was dried in vacuum. ^1^H-NMR (400 MHz, 0.2M NaOD in D_2_O) δppm: 3.64 + 3.55 (2 × dd, 2H, -CH_2_OH), 3.40 (m, 1H -CHNH_3_^+^), 3.10 + 2.90 (2 × dd, 2H, -CH_2_SO_3_^−^), ^13^C-NMR (400 MHz, 0.2M NaOD in D_2_O) δppm: 64.0 (-CH_2_OH), 53.0 (CH_2_SO_3_^−^), 49.2 (-CHNH_3_^+^) (ESI-MS (positive) *m/z* 156.03232 [M + H]^+^.

## 4. Conclusions

In this work, we describe the identification and quantification of cysteinolic acid, which underline its importance as an algal osmolyte. Cysteinolic acid is widespread among different phyla of phytoplankton and is an abundant highly polar compatible solute.

## Figures and Tables

**Table 1:** Quantitative survey of cysteinolic acid and other osmolytes in xenic marine microalgae at 35 PSU. Replicates: *N* = 3, error based on standard deviations. A plus (+) indicates the presence of a signal below the limit of quantification, a minus (−) indicates the absence of the correspondent signal. Cell volumes of *T. weissflogii* for determination of intracellular cysteinolic acid concentration were obtained from Fenizia et al. 2020 [7]; cell volumes of the other species were based microscopy measurement and calculation according to their geometric shape [39]. Data for DMSOP from Thume et al. 2018 [11].

Species	DMSA	Gonyol	GBT (Fmol Cell^−1^)	Homarine (Fmol Cell^−1^)	DMSP (Fmol Cell^−1^)	DMSOP (Fmol Cell^−1^)	Ectoine (Fmol Cell^−1^)	Cysteinolic Acid (Fmol Cell^−1^)	Cysteinolic Acid (mM)
*Prorocentrum minimum*	+	+	37.9 ± 4.8	0.27 ± 0.06	463.4 ± 52.6	3.66 ± 1.23	42.0 ± 6.1	50.6 ± 8.1	71.1 ± 11.4
*Prymnesium parvum*	−	+	1.5 ± 0.7	0.04 ± 0.02	47.4 ± 5.3	0.029 ± 0.005	4.8 ± 1.2	1.9 ± 0.8	17.9 ± 7.3
*Amphidinium carterae*	+	+	+	0.09 ± 0.04	+	−	9.1 ± 1.5	17.2 ± 2.8	18.5 ± 3.0
*Thalassiosira pseudonana*	+	−	7.7 ± 0.9	2.0 ± 0.2	4.8 ± 0.6	−	1.9 ± 0.2	2.8 ± 0.3	17.5 ± 2.0
*Skeletonema costatum*	−	−	+	0.06 ± 0.01	28.5 ± 4.1	0.029 ± 0.005	10.0 ± 1.5	10.9 ± 0.5	42.2 ± 2.1
*Emiliania huxleyi*	−	+	0.73 ± 0.04	0.41 ± 0.03	7.3 ± 0.7	0.029 ± 0.013	0.54 ± 0.06	1.0 ± 0.1	11.9 ± 0.7
*Isochrysis galbana*	−	+	4.8 ± 0.2	1.3 ± 0.1	13.8 ± 0.6	0.017 ± 0.003	1.6 ± 0.1	1.0 ± 0.03	10.8 ± 0.4
*Thalassiosira weissflogii*	−	−	234.7 ± 35.7	35.4 ± 2.5	−	−	85.2 ± 13.1	22.3 ± 11.3	8.0 ± 4.0

## Data Availability

The underlying data are deposited in the zenodo repository under the following link: https://doi.org/10.5281/zenodo.5697964 (accessed on 25 November 2021).
